# The evaluating self-management and educational support in severely obese patients awaiting multidisciplinary bariatric care (EVOLUTION) trial: principal results

**DOI:** 10.1186/s12916-017-0808-6

**Published:** 2017-03-02

**Authors:** Raj S. Padwal, Scott Klarenbach, Arya M. Sharma, Miriam Fradette, Susan E. Jelinski, Alun Edwards, Sumit R. Majumdar

**Affiliations:** 1 Department of Medicine, University of Alberta, Edmonton, Alberta Canada; 2 Alberta Diabetes Institute, Edmonton, Alberta Canada; 3 Digestive Health Strategic Clinical Network, Alberta Health Services, Calgary, Alberta Canada; 4 Department of Medicine, University of Calgary, Calgary, Alberta Canada; 5 Raj Padwal, 5-134A Clinical Sciences Building, 11350-83rd Ave, Edmonton, Alberta T6G 2B7 Canada

**Keywords:** Bariatric care, Wait list, Severe obesity, Randomized controlled trial, Self-management

## Abstract

**Background:**

In Canada, demand for multidisciplinary bariatric (obesity) care far outstrips capacity. Consequently, prolonged wait times exist that contribute to substantial health impairments. A supportive, educational, self-management intervention (with in-person and web-based versions) for patients wait-listed for bariatric care has already been implemented in Northern and Central Alberta, Canada, but its effectiveness is unknown. The objective of this trial is to evaluate the clinical and economic outcomes of two self-management programs of varying intensity that are currently in use.

**Methods:**

We conducted a pragmatic, prospective, parallel-arm, randomized controlled trial of 651 wait-listed patients from two regional bariatric programs. Patients were randomized to (1) an in-person, group-based intervention (13 sessions; *n* = 215) or (2) a web-based intervention (13 modules; *n* = 225) or (3) control group (printed educational materials; *n* = 211). After randomization, subjects had 3 months to review the content assigned to them (the intervention period) prior to bariatric clinic entry. The primary outcome was the proportion of patients achieving 5% weight loss at 9 months. Intention-to-treat two-way comparisons were performed and adjusted for baseline age, sex, site and body mass index.

**Results:**

At baseline, mean age was 40.4 ± 9.8 years, mean weight was 134.7 ± 25.2 kg, mean body mass index was 47.7 ± 7.0 kg/m^2^ and 83% of participants were female. A total of 463 patients (71%) completed 9 months follow-up. At least 5% weight loss was achieved by 24.2% of those in the in-person strategy, 24.9% for the web-based strategy and 21.3% for controls (adjusted *p* value = 0.26 for in-person vs. controls, 0.28 for web-based vs. controls, 0.96 for in-person vs. web-based). Absolute and relative (% of baseline) mean weight reductions were 3.7 ± 7.1 kg (2.7 ± 5.4%) for in-person strategy, 2.8 ± 6.7 kg (2.0 ± 4.8%) for web-based and 2.9 ± 8.8 kg (1.9 ± 5.9%) for controls (*p* > 0.05 for all comparisons). No between-group differences were apparent for any clinical or humanistic secondary outcomes. Total annual costs in Canadian dollars were estimated at $477,000.00 for the in-person strategy, $9456.78 for the web-based strategy and $2270.31 for provision of printed materials.

**Discussion:**

Two different self-management interventions were no more effective and were more costly than providing printed education materials to severely obese patients. Our findings underscore the need to develop more potent interventions and the importance of comprehensively evaluating self-management strategies before widespread implementation.

**Trial registration:**

ClinicalTrials.gov, NCT01860131. Registered 17 May 2013.

**Electronic supplementary material:**

The online version of this article (doi:10.1186/s12916-017-0808-6) contains supplementary material, which is available to authorized users.

## Background

Severe obesity (defined as a body mass index (BMI) ≥40 kg/m^2^) currently affects 8% of Americans and 3% of Canadians and is the most rapidly growing obesity subclass [1, 2]. Severe obesity increases the risk of type 2 diabetes mellitus by more than eightfold [3]; reduces life expectancy by 5–13 years [4]; increases health care expenditures by 50–200% [5]; and dramatically reduces quality of life [6].

In Canada’s publicly funded, universally accessible health care system, bariatric care for severely obese individuals is delivered primarily within a small number of multidisciplinary bariatric specialty clinics located in major cities [7]. Upon entry, patients receive intensive lifestyle modification (diet, exercise, behavioural modification) counselling; in addition, after undergoing multidisciplinary assessment to determine suitability, highly selected patients undergo bariatric surgery. As a result of the limited number of clinics and large number of patients, demand-supply gaps for multidisciplinary bariatric care exist and have resulted in protracted multiyear wait times [8, 9]. In other countries with publicly funded health care, such as the United Kingdom, waits are similarly prolonged [10–12]. Although, on average, the weight and health comorbidities of wait-listed patients appear stable over 2 years, baseline health status is markedly impaired compared to population norms [13–15]. Most wait-listed patients report that waiting contributes to physical, mental and financial deterioration over time, and nearly 75% are interested in receiving supportive care as they wait [16].

To try and meet this unmet patient-centred demand, prime wait-listed patients on the basics of weight management and better support wait-listed patients facing protracted wait times in Alberta, Canada, Alberta Health Services (AHS) developed a multidisciplinary, in-person, group-based educational self-management program. This in-person program was delivered in community centres across Edmonton (Northern Alberta) beginning in 2008 and expanded to other centres within the province over the next 4–5 years. In 2012, a less costly web-based version of the program, with similar content, was initiated in the bariatric program in Calgary (Southern Alberta).

Prior to implementation, neither program had been formally and rigorously evaluated in terms of effectiveness or cost-effectiveness. Therefore, we performed a randomized controlled trial (RCT) to evaluate the benefits and costs of both the in-person and web-based programs compared to a printed educational materials control group. Thus, the primary rationale for this trial was to verify that scarce medical resources were being optimally allocated and, in addition, to determine the most efficient method of delivering self-management support.

## Methods

A detailed trial protocol has been published previously [17]. All subjects provided written informed consent. The evaluating self-management and educational support in severely obese patients awaiting multidisciplinary bariatric care (EVOLUTION) trial protocol was approved by the University of Alberta Research Ethics Board (PRO00031699) and, prior to patient enrolment, the trial was formally registered at ClinicalTrials.gov (NCT01860131).

### Design

We conducted a 9-month pragmatic, prospective, RCT enrolling consecutive, consenting patients with severe obesity newly wait-listed for adult bariatric specialty care. The study was conducted in Edmonton and Red Deer (Northern and Central Alberta, respectively). Patients were randomly assigned (1:1:1) to one of three groups:

1. In-person, community-based self-management
2. Web-based self-management
3. A one-time provision of printed educational materials

Computer-generated randomization was performed centrally and independently by the EPICORE centre (www.epicore.ualberta.ca) to ensure allocation concealment from all research personnel; randomization was stratified by participating study centre.

### Setting

The Edmonton Weight Wise program, established in 2005, was the first large-scale, multidisciplinary bariatric program in Alberta. Weight Wise delivers integrated, patient-focused, evidence-based care to the Edmonton Zone of Alberta Health Services (AHS). Treatments are guideline-concordant and based on the Canadian obesity guidelines [18]. This region is one of the largest integrated health delivery systems in Canada, serves a catchment population of 1.6 million residents and has an annual health care budget of 2 billion dollars.

Weight Wise includes a central, region-wide, single-point-of-access referral system. The Adult Specialty Clinic offers intensive multidisciplinary medical/surgical bariatric care to patients with BMI levels of ≥35 kg/m^2^ (estimated to be at least 125,000 individuals within the region). Patients are referred for both medical and surgical management. About 64% of wait-listed patients are interested in bariatric surgery, and the remainder in medical management alone. All patients receive medical therapy initially and for several months before evaluation for surgery (if interested). The decision to perform surgery depends, in part, on how much effort and commitment the patient has put into lifestyle modification (even if unsuccessful). Therefore, patients interested in surgery cannot forgo medical weight loss recommendation adherence because they wish to have surgery.

At the time that this trial was performed, approximately 1200 new referrals were being processed annually, and 300 bariatric surgeries were being performed. Wait times have fluctuated and varied from as short as 4 months to as long as 30 months. Within the Weight Wise program, nurses are case managers and are responsible for coordinating care with other health care providers. Dieticians provide the bulk of the health behaviour modification counselling. This is reinforced by other care providers (physician, sleep specialist, psychologist, psychiatrist, occupational therapist, physiotherapist and social worker), who see patients for targeted indications within their area of specialization. The program in Red Deer was modelled after the parent Weight Wise program. Red Deer is a smaller centre that serves a more rural population, with approximately 600 referrals and 100 bariatric surgeries per year.

### Interventions

#### In-person self-management arm

This program varied slightly over the trial period, but, at its core, broadly consisted of 13 sessions (Additional file 1: Table S1) delivered in a group format by a multidisciplinary team at community health centres in Edmonton and Red Deer. Each session was approximately 2.5 hours long and was led by the appropriate content expert(s) from a team of four registered nurses, one Canadian Society for Exercise Physiology (CSEP) specialist, one MSc psychologist and one registered dietician. The program is free of charge and has been designed to educate patients regarding proper diet and exercise; improve their weight management skills by enhancing self-management and self-efficacy; and help them identify/overcome barriers to success. In addition to providing supportive care to wait-listed patients, the program is intended to prime patients for weight management success once they begin bariatric care. The evidence-based curriculum [18] stresses healthful eating; gives practical tips to increase physical activity; teaches basic behavioural modification techniques such as goal setting and self-monitoring; and includes strategies for dealing with stress and maladaptive eating behaviours such as emotional eating.

#### Web-based self-management arm

The web-based program was very similar in content to the in-person intervention (Additional file 1: Table S2), with the exception that it was delivered solely in an online format. Each patient had a personalized login that was tracked by the study team. All 13 modules were available to the subject on a single online platform, accessible any time after randomization. Subjects were asked to read all 13 modules (Additional file 1: Table S2) over a 3-month period.

#### Control arm

Controls received a one-time provision of printed educational materials at the time of enrolment, consisting of Canada’s Guide to Healthy Living and Canada’s Physical Activity Guide. These materials are guideline-based, user-friendly and visually appealing. They are produced by the Canadian government and made available free of charge.

Of note, both for purposes of ensuring fidelity and maintaining some degree of ’attention control’, subjects in all three study arms were contacted by telephone 1 week after enrolment for troubleshooting and to encourage them to read the printed materials, register for in-person sessions or access the web modules.

### Participants

We enrolled patients with BMI levels ≥35 kg/m^2^ who were newly wait-listed for adult (age >18 years) bariatric specialty care at the Edmonton or Red Deer clinic. Patients with one or more of the following characteristics were excluded: (1) completed more than four Weight Wise Community Modules (web-based or group session) in previous 3 months, (2) pregnant, (3) unable to read/write/comprehend English, (4) unable to access the web, (5) unable or unwilling to attend in-person sessions, (6) uncontrolled severe personality disorder, active psychosis, active substance dependence and/or major cognitive impairment, (7) deemed unsuitable by the study investigators, (8) participated in concurrent trial related to obesity management, (9) resided more than 1 hour driving time away from Weight Wise clinic or (10) declined to participate.

### Enrolment procedures and timelines

Consecutive, consenting, eligible patients were enrolled between 2013 and 2015. Study participants were instructed to not begin any other new lifestyle modification or weight loss-related interventions during the first 3 months of the study. Those randomized to the in-person and web-based interventions were instructed to complete the entire program within a 3-month period. Thereafter, all subjects, including controls, immediately entered the bariatric clinic and commenced multidisciplinary bariatric care. This meant that the usual wait-listed period for entry into the Weight Wise clinic was waived for trial participants. Subjects were followed for an additional 6 months after entry into the clinic. Thus, overall, the trial consisted of a 3-month period during which the interventions were applied, followed by a 6-month follow-up period, with ascertainment of outcomes at baseline, 3 months, 6 months and 9 months (see below). To prevent between-arm contamination, subjects received specific information on their assigned treatment group only. Access to the web-based program was controlled using a hidden URL, which was given only to participants randomized to the web-based intervention arm, and patients in the web-based or control groups were asked not to attend the in-person community sessions.

### Outcomes

Data collection procedures including detailed case report forms have been published previously [17]. Although clinic staff could not be blinded to allocation status, all outcome assessments were performed by research assistants working independently from regular clinic staff. Data were collected at baseline and at 3, 6 and 9 months post-randomization, with the primary analysis focusing on 9-month outcomes.

#### Anthropometric

Body weight was measured using a validated, calibrated Scale-Tronix bariatric scale and recorded to the nearest 0.1 kg, with the subject wearing light indoor clothing with empty pockets, no shoes and an empty bladder. Height was measured to the nearest 0.1 cm using a wall-mounted stadiometer. Blood pressure was measured with a Microlife Watch BP automated monitor, with three readings taken simultaneously in each arm, the first reading discarded and the latter two averaged. The arm that had the highest mean blood pressure at the baseline visit was used to calculate the 9-month blood pressure change.

#### Clinical

The primary outcome was the proportion of patients achieving 5% weight loss, considered a clinically important degree of weight loss by experts and contemporary guidelines [18]. Absolute and relative weight loss and BMI change were also assessed. Additional clinical outcomes included change in blood pressure, fasting lipids and A1c and the change in prevalence of hypertension, diabetes and dyslipidemia as previously described. Hypertension was considered present if self-reported, if blood pressure levels were ≥140/90 mm Hg or if antihypertensive medications were prescribed. Diabetes was defined based upon self-report, a baseline A1c ≥6.5% and/or antidiabetic drug therapy. Dyslipidemia was diagnosed in the presence of one of the following: self-report, treatment with a lipid-lowering agent or an abnormality on the baseline fasting lipid panel (low-density lipoprotein (LDL) cholesterol ≥5.2 mmol/L, high-density lipoprotein (HDL) cholesterol <0.9 mmol/L or triglyceride level ≥2.8 mmol/L).

To ensure that utilization was equivalent once patients entered the clinic, we tracked the mean number of clinic visits to each type of health care provider over the follow-up period.

#### Humanistic

We used previously validated instruments to assess health-related quality of life (Short Form Survey (SF-12) [19] and EuroQol five dimensions (EQ-5D) [20]), preference-based utilities [20], satisfaction with medical care (Patient Satisfaction Questionnaire (PSQ) [21] scored on a 5-point Likert scale), self-efficacy (Weight Efficacy Life-Style Questionnaire (WEL) [22], depression (Patient Health Questionnaire (PHQ-8) [23] and readiness to change (assessed using a visual analogue scale (VAS) ranging from 0 to 10).

#### Resource use and costs for each study arm

The overall annual and per-patient costs of the two interventions relative to the control group were calculated using a methodology conforming to the three-step micro-costing technique of identification, measurement and valuation of resources [24, 25]. Estimates of health care professional time for creation and delivery of the interventions were obtained from AHS Chronic Disease program managers. In 2012–2013, 1707 patients were referred to the Edmonton and Red Deer programs and had an initial consult; for costing it was assumed that annually 1707 patients would receive the in-person strategy or the web-based strategy or be mailed printed educational materials.

The health care professional cost to develop and update the materials used in the in-person and web-based study arms as well as delivery of the in-person strategy were determined by obtaining estimates of time required and use of AHS wage rates for each category of staff (nurses, dietician, exercise therapist, psychologist). The resources to develop both the web-based and in-person interventions consisted primarily of the health care professional time required for literature review, summarizing the content and preparation of the content for delivery. The one-time development cost was amortized over a 5-year period. For the in-person strategy, a registered dietician and registered nurse performed the bulk of the work, with smaller contributions from an exercise specialist and a psychologist. No overhead costs were assigned to the in-person strategy, as sessions were conducted in health care facilities at off-peak hours. A registered dietician developed the web-based modules.

Further, both the in-person sessions and web-based modules were updated every 2 years; therefore, the health care professional time required for this work was estimated and included in the overall costs. Finally, the costs of delivering content, including generating patient lists and mailing out instructions in the web-based group as well as hosting and delivering in-person sessions, were estimated. Current wage rates in Alberta, Canada for each type of health care professional delivering content were used. Hourly wage rate assumptions were $42.62 for a registered dietician, $42.62 for a registered nurse, $44.44 for an exercise specialist and $46.88 for a psychologist.

Resources required for the control arm included patient list generation, administrative work required to prepare and address the envelopes and mailing costs [26]. Costs associated with development of the materials were not included; the mailed literature (Canada’s Food Guide) is provided free of cost by Health Canada.

All costs are reported in 2013 Canadian dollars.

### Statistical analyses

Descriptive analyses were performed including calculation of means, medians and standard deviations (SDs). Baseline variables were compared between the three study groups using one-way analysis of variance (ANOVA) for continuous outcomes and chi-squared tests for dichotomous ones.

Between-group change scores were compared using multivariable logistic regression for dichotomous variables (including the primary) and linear regression for continuous outcomes, adjusting for age, sex, site and baseline BMI. An intention-to-treat analysis was performed using a baseline-observation-carried-forward approach, designated a priori [17], for the primary analysis. A completers analysis, limited to those participants with full baseline and 9-month data, was also reported for weight and BMI-based outcomes. Multiple imputation was not performed because the data were not missing completely at random [27]. The primary outcome comparisons of interest were between the in-person strategy and controls and the online strategy and controls. Subsequently, the in-person and online interventions were then compared.

One-way ANOVA was used to compare health care provider visit frequency and costs across study arms, with *t* tests used to compare between-study arms if significant differences were found. We considered *p* values less than 0.05 statistically significant. No adjustment for multiple testing was performed [28]. All analyses were performed using SAS® (Version 9.3, Cary, NC, USA).

#### Sample size estimate

The study was powered to detect a 15% difference between the two interventions and controls in the proportion of 5% weight responders with an alpha level of 0.05 and a power of 0.90. We assumed that the control arm would result in a 5% weight loss in 20% of subjects (i.e. the control event rate = 20% at one year) [29]. The initial sample size estimate was for ~180 patients per arm or 540 patients total. This was adjusted upwards to account for potential attrition and permit secondary and subgroup analyses to arrive at the final sample size.

## Results

**Fig. 1:**
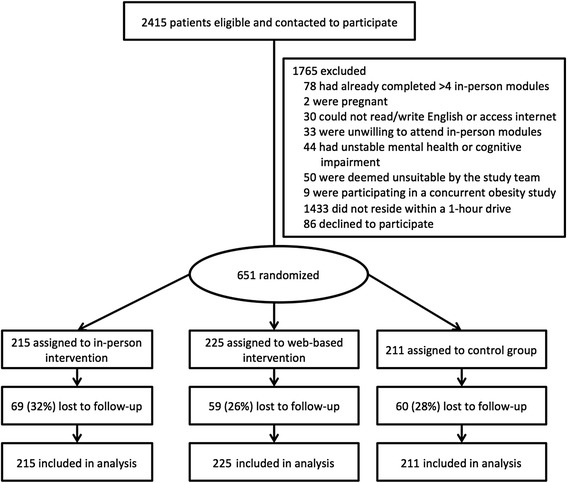
Study overview

Of 2416 patients contacted, 1765 (73%) were excluded (Fig. 1). The primary reason for exclusion was that the patient did not reside in the study locality (within a 1-hour drive; *n* = 1433 (59%)). In total, 651 patients were enrolled and randomized to the in-person strategy (*n* = 215), web-based strategy (*n* = 225) or the control arm (*n* = 211).

### Baseline characteristics

Baseline characteristics are summarized in Table 1. In the overall study sample, mean age was 40.4 ± 9.8 years, mean weight was 134.7 ± 25.2 kg, mean BMI was 47.7 ± 7.0 kg/m^2^ and 83% of participants were female. There were few clinically meaningful differences across study arms (Table 1).

**Table 1:** Baseline characteristics

Variable	Overall *n* = 651	In-person *n* = 215	Web-based *n* = 225	Controls *n* = 211	*p* value (among groups)*
Age (years, mean ± SD)	40.4 ± 9.8	40.5 ± 9.9	40.6 ± 10.1	40.4 ± 9.3	0.9
Weight (kg, mean ± SD)	134.7 ± 25.2	131.1 ± 25.9	134.4 ± 23.5	138.8 ± 25.8	0.01
BMI (kg/m^2^ ± SD)	47.7 ± 7.0	46.7 ± 7.4	47.7 ± 6.6	48.7 ± 7.2	0.01
Sex (Females, %)	540 (83)	176 (81)	183 (81)	181 (86)	0.4
White race (%)	594 (91)	195 (90)	205 (91)	194 (92)	0.9
Education, no. (%)					
Some high school or less	52 (7.9)	19 (8.8)	19 (8.4)	14 (6.6)	
High school diploma	110 (16.9)	29 (13.5)	43 (19.1)	38 (18.0)	
Some post-secondary	118 (18.1)	38 (17.7)	38 (16.9)	42 (19.9)	
Completed post-secondary	369 (56.7)	129 (60)	123 (54.7)	117 (55.5)	0.4
Smoking status, no. (%)					
Current smoker	84 (12.9)	25 (11.6)	33 (14.7)	26 (12.3)	
Former smoker	143 (22.0)	47 (21.9)	49 (21.8)	47 (22.3)	
Never smoker	424 (65.1)	143 (66.5)	143 (63.6)	138 (65.4)	0.9
Medical comorbidities, no. (%)					
Hypertension	346 (53.2)	106 (49.3)	113 (50.2)	127 (60.2)	0.04
Diabetes	162 (24.9)	52 (24.2)	61 (27.1)	49 (23.2)	0.6
Dyslipidemia	274 (42.1)	91 (42.3)	92 (40.4)	92 (43.6)	0.8
Sleep apnea	180 (27.7)	53 (24.7)	78 (34.7)	49 (23.2)	0.01
Cardiovascular event	15 (2.3)	3 (1.4)	6 (2.7)	6 (2.8)	0.5
Mental illness	319 (49.0)	103 (47.9)	105 (46.7)	111 (52.6)	0.4
Clinical measurements, mean ± SD					
Systolic BP (mmHg) (*n* = 568)	136.1 ± (17.1)	135.4 ± (15.1)	134.0 ± (14.9)	139.0 ± (20.5)	0.01
Diastolic BP (mmHg) (*n* = 569)	82.5 ± 12.5	81.6 ± 10.6	81.5 ± 11.3	84.3 ± 14.8	0.05
Total cholesterol (mmol/L) (*n* = 586)	4.7 ± 0.9	4.7 ± 0.9	4.8 ± 0.9	4.7 ± 1.0	0.9
HDL cholesterol (mmol/L) (*n* = 586)	1.2 ± 0.3	1.2 ± 0.3	1.2 ± 0.3	1.2 ± 0.3	0.2
LDL cholesterol (mmol/L) (*n* = 576)	2.7 ± 0.8	2.7 ± 0.8	2.7 ± 0.8	2.7 ± 0.8	1.0
Triglycerides (mmol/L) (*n* = 586)	1.8 ± 0.9	1.8 ± 1.0	1.8 ± 0.9	1.7 ± 0.8	0.9
A1c (%) (*n* = 573)	6.0 ± 1.2	6.0 ± 1.1	6.0 ± 1.4	5.9 ± 1.1	0.7
EQ-5D VAS score	53.1 ± 18.9	54.3 ± 18.4	54.6 ± 18.6	50.3 ± 19.5	0.03
EQ-5D index score	0.84 ± 0.06	0.83 ± 0.06	0.84 ± 0.06	0.84 ± 0.06	0.33
SF-12 PC score	37.5 ± 8.8	38.0 ± 8.9	37.3 ± 8.7	37.2 ± 9.0	0.58
SF-12 MC score	39.9 ± 9.6	40.6 ± 9.6	40.2 ± 9.6	39.0 ± 9.7	0.24
WEL score	109.5 ± 35.4	108.3 ± 36.2	114.9 ± 33.2	104.9 ± 36.0	0.01
Readiness-to-change score	9.0 ± 1.1	8.9 ± 1.1	9.0 ± 1.1	8.9 ± 1.2	0.58
PHQ-8 score	17.4 ± 5.5	17.4 ± 5.4	17.1 ± 5.6	17.7 ± 5.4	0.58

*Using ANOVA for continuous variables and chi-square for dichotomous variables

*MC* mental component, *PC* physical component

### Follow-up and attendance to the interventions

Follow-up to 9 months was completed in 463 participants (71%). Of the 188 patients (29%) who withdrew early, 68 no longer wished to participate in the study, 94 were lost to follow-up and 26 left for other reasons. No deaths occurred during the study period. Early withdrawal occurred in 69 patients (32%) randomized to the in-person strategy, 59 patients (26%) randomized to the web-based strategy and 60 patients (28%) randomized to the control arm. No differences in health care provider visit frequency over the follow-up period were apparent across study arms after randomization (Additional file 1: Table S3; *p* > 0.05 for all comparisons).

Within the 3-month window, 75% of subjects assigned to the in-person strategy attended at least one session, with 62% attending three or more sessions. The mean number of attended in-person sessions was 5.3 ± 4.1. One patient randomized to each of the web-based or control arms reported attending an in-person session. For subjects assigned to the web-based strategy, 86% completed at least one module, 80% completed three or more modules and the mean number of completed modules was 9.2 ± 4.9. No subjects assigned to the in-person or control arms reported viewing a web-based module.

### Weight and BMI changes

Anthropometric outcomes are summarized in Table 2. The proportion of patients achieving 5% weight loss was 24.2% for the in-person strategy, 24.9% for the web-based strategy and 21.3% in controls (adjusted *p* value = 0.26 for in-person vs. controls, 0.28 for web-based vs. controls, 0.96 for in-person vs. web-based). Absolute and relative (% of baseline) mean weight reductions were 3.7 ± 7.1 kg (2.7 ± 5.4%) for the in-person strategy (*n* = 215), 2.8 ± 6.7 kg (2.0 ± 4.8%) for the web-based strategy and 2.9 ± 8.8 kg (1.9 ± 5.9%) for controls (*p* > 0.05 for all comparisons).

**Table 2:** Changes in weight and BMI

Outcome	In-person	Web-based	Controls	In-person minus controls (95% CI)	*p* value*	Web-based minus controls (95% CI)	*p* value*	In-person minus web-based (95% CI)	*p* value*
Baseline-observation-carried-forward analysis									
	*n* = 215	*n* = 225	*n* = 211						
5% weight loss responders, %	24.2	24.9	21.3	2.9 (-5.1 to 10.9)	0.26	3.6 (-4.4 to 11.5)	0.28	-0.7 (-8.7 to 7.3)	0.96
Absolute weight change, kg ± SD	-3.7 ± 7.1	-2.8 ± 6.7	-2.9 ± 8.8	-0.8 (-2.3 to 0.8)	0.16	0.2 (-1.3 to 1.6)	0.96	-0.9 (-2.2 to 0.4)	0.11
Percent weight change, % ± SD	-2.7 ± 5.4	-2.0 ± 4.8	-1.9 ± 5.9	-0.9 (-1.9 to 0.2)	0.08	-0.1 (-1.1 to 0.93)	0.79	-0.8 (-1.7 to 0.2)	0.09
BMI change, kg/m^2^ ± SD	-1.3 ± 2.5	-1.0 ± 2.4	-1.0 ± 3.0	-0.3 (-0.8 to 0.2)	0.13	0 (-0.5 to 0.5)	0.88	-0.3 (-0.8 to 0.1)	0.12
Completers analysis									
	*n* = 141	*n* = 164	*n* = 149						
5% weight loss responders, %	36.9	34.2	30.2	6.7 (-4.2 to 17.6)	0.13	3.9 (-6.4 to 14.3)	0.33	2.7 (-8.1 to 1.4)	0.58
Absolute weight, kg ± SD	-5.6 ± 8.2	-3.8 ± 7.6	-4.2 ± 10.2	-1.4 (-3.6 to 0.7)	0.13	0.4 (-1.6 to 2.3)	0.88	-1.8 (-3.6 to -0.06)	0.04
Percent weight change, % ± SD	-4.2 ± 6.2	-2.7 ± 5.4	-2.7 ± 6.8	-1.5 (-3.0 to -0.01)	0.046	0 (-1.4 to 1.4)	0.86	-1.5 (-2.8 to -0.2)	0.02
BMI change, kg/m^2^ ± SD	-2.0 ± 2.8	-1.4 ± 2.7	-1.4 ± 3.5	-0.6 (-1.3 to 0.1)	0.08	0.1 (-0.6 to 0.7)	0.92	-0.6 (-1.3 to -0.01)	0.04

*Adjusted for baseline age, sex and baseline BMI using logistic or linear regression

*CI* confidence interval, *SD* standard deviation, *BMI* body mass index

Results of the completers sensitivity analyses were broadly consistent with the primary analysis, with no statistically significant differences between study arms for the primary endpoint (Table 2). Percent weight loss was slightly greater in patients receiving the in-person intervention relative to controls (4.2% vs. 2.7%; adjusted *p* = 0.046). Absolute weight loss, relative weight loss and BMI reductions were modestly, but statistically significantly, higher in the in-person arm relative to the web-based strategy (Table 2).

### Secondary clinical and humanistic endpoints

No clinically important or statistically significant differences in any of the secondary endpoints were observed between study arms (Table 3).

**Table 3:** Changes in secondary endpoints

Outcome	Sample size	In-person	Web-based	Controls	In-person minus controls	*p* value*	Web-based minus controls	*p* value*	In-person minus web-based	*p* value*
Systolic BP (mm Hg)	568	-2.2 ± 10.0	-0.7 ± 10.3	-2.6 ± 15.4	0.4 ± 12.9	0.79	1.8 ± 13.0	0.20	-1.4 ± 10.1	0.17
Diastolic BP (mm Hg)	569	-1.1 ± 8.6	-1.6 ± 8.6	-1.9 ± 11.8	0.8 ± 10.3	0.56	0.3 ± 10.3	0.82	0.5 ± 8.6	0.57
Total cholesterol (mmol/L)	586	-0.1 ± 0.7	-0.1 ± 0.6	-0.2 ± 0.6	0.1 ± 0.6	0.20	0.02 ± 0.7	0.86	0.1 ± 0.6	0.19
HDL cholesterol (mmol/L)	586	0.01 ± 0.14	0.01 ± 0.16	-0.01 ± 0.13	0.02 ± 0.13	0.11	0.02 ± 0.14	0.18	0.003 ± 0.15	0.8
LDL cholesterol (mmol/L)	576	-0.1 ± 0.4	-0.05 ± 0.4	-0.09 ± 0.4	-0.01 ± 0.4	0.77	0.04 ± 0.4	0.34	-0.05 ± 0.4	0.24
Triglycerides (mmol/L)	586	1.8 ± 1.0	1.8 ± 0.9	1.7 ± 0.8	0.04 ± 0.55	0.50	0.05 ± 0.58	0.38	0.1 ± 0.7	0.15
A1c (%)	573	-0.10 ± 0.40	-0.16 ± 0.80	-0.11 ± 0.50	0.01 ± 0.45	0.79	-0.05 ± 0.64	0.54	0.06 ± 0.63	0.38
EQ-5D VAS score	647	10.3 ± 15.7	11.4 ± 17.3	11.0 ± 17.8	0.7 ± 16.8	0.74	0.4 ± 17.5	0.92	-1.0 ± 16.6	0.52
EQ-5D index score	645	0.03 ± 0.05	0.02 ± 0.04	0.02 ± 0.05	0.01 ± 0.05	0.11	0.001 ± 0.05	0.88	0.006	0.15
SF-12 PC score	639	4.1 ± 7.3	4.9 ± 6.7	3.7 ± 7.2	0.3 ± 7.2	0.63	1.2 ± 6.9	0.08	-0.9 ± 7.0	0.21
SF-12 MC score	639	4.2 ± 8.1	4.1 ± 7.4	4.7 ± 8.8	-0.5 ± 8.5	0.67	-0.6 ± 8.1	0.48	0.06 ± 7.8	0.99
WEL score	649	22.3 ± 31.2	20.8 ± 29.7	22.2 ± 34.6	0.1 ± 32.9	0.90	-1.4 ± 32.1	0.59	1.5 ± 30.4	0.58
Readiness-to-change score	650	-0.08 ± 1.19	-0.14 ± 1.10	-0.07 ± 1.30	0.003 ± 1.30	0.95	0.06 ± 1.20	0.64	0.06 ± 1.13	0.54
PHQ-8 score	632	-2.8 ± 4.4	-3.1 ± 4.2	-3.1 ± 4.8	0.3 ± 4.6	0.63	0.04 ± 4.5	0.93	0.3 ± 4.3	0.54

Numbers are mean ± SD. Baseline-observation-carried-forward imputation

*Adjusted for baseline age, sex and baseline BMI using logistic or linear regression

*BP* blood pressure, *CI* confidence interval, *EQ* EuroQol, *HDL* high-density lipoprotein, *LDL* low-density lipoprotein, *MC* mental component, *PC* physical component, *PHQ* Patient Health Questionnaire, *SD* standard deviation, *SF* Short Form, *VAS* visual analogue scale, *WEL* Weight Efficacy Lifestyle questionnaire

### Costs

#### In-person strategy

A total time of 535 hours was required for initial development of the in-person strategy content, at a total cost of $23,128. Two-year content updates required an additional 125 hours, accruing a cost of $10,998 for each update cycle. Delivery of each session took approximately 4 hours of health care professional time, which included 2.5 hours of content delivery, 0.5 hour of pre-session preparation and post-session question-and-answer time and 1 hour of travel time. Although in-person sessions had a maximum capacity of 22 patients, they were delivered, on average, to 8.2 patients. The total annual cost of delivery of a full cycle of the in-person intervention (all 13 sessions each at 4 hours) was $2241.

Per-patient development and update costs were estimated at $2.71 and $3.22, respectively. Assuming 8.2 patients attend each set of modules, the per-patient cost of delivery of the in-person strategy was $273.40. The total per patient cost (per year) was $279.33, and the total annual cost was $476,816.31.

#### Web-based strategy

An estimated 403 hours of registered dietician’s time was required for web-based module development, with a total cost of $17,175. Estimated costs for 2-year updates were $8072 for an additional 189 hours of work. Annual web hosting and information technology support costs were estimated at $2000.

For 1707 patients, per-person costs for initial development, update and hosting were $2.01, $2.36 and $1.17, respectively, and the overall web-based strategy average per-patient cost was $5.54. The total annual cost of this strategy was $9456.78.

#### Controls

The estimated per-person cost for mailing the printed educational materials was $1.33 per person, with a total annual cost of $2270.31.

#### Costs of each intervention relative to controls

Relative to the control arm, the per-patient incremental cost was $278 for the in-person strategy and $274 for the web-based strategy. Total incremental annual costs were $474,546 for the in-person strategy and $7188 for the web-based strategy.

## Discussion

To summarize, in this randomized controlled trial of more than 600 severely obese patients enrolled in a large Canadian bariatric program, more intensive self-management support interventions, delivered either in-person or online, were no more effective (and yet more costly) than a one-time intervention that consisted of providing printed educational materials to patients. In particular, in the primary analysis, neither active intervention effectively reduced weight, optimized cardio-metabolic parameters or improved humanistic endpoints compared with controls. Although some statistically significant differences were found between study arms in the completers analysis, these were modest, subject to confounding by healthy adherer effects, would not be considered clinically important and were of marginal significance given that we did not adjust for multiple testing.

In aggregate, the patients in our trial had substantially impaired physical and mental health, poor self-efficacy and (according to the PHQ-8) on average had symptoms consistent with major depression. These self-management strategies were developed to mitigate this degree of suffering, improve health and quality of life, help patients cope with extended bariatric wait times and ready patients for more intensive medical and surgical multidisciplinary care [17]. Our findings indicate that these strategies are not effective and, thus, alternative methods of supporting these patients will need to be considered (but should also be rigorously evaluated to ensure that they are worthwhile prior to implementation).

In terms of the broader literature, a recent systematic review of reviews examining the effectiveness of self-directed interventions for weight loss, whether web-based or conventional, judged only 7 of 20 reviews to be of high quality [30]. Even when statistically significant reductions in weight were found, the degree of weight loss was very modest (e.g. 1.5 kg) and of a clinically unimportant magnitude [31], with few reviews linking intervention content to effectiveness. Similarly, a recent systematic review and meta-analysis of 84 studies examining e-Health interventions reported very modest weight loss compared to minimal intervention controls (mean incremental weight reduction of 1.40 kg (95% CI 0.82–1.98)) [32]. Furthermore, an 818-patient recent cluster randomized controlled trial done in 56 primary care practices England found that in-person and web-based interventions were only minimally effective, producing statistically significant (but by our prespecified trial criteria, clinically inconsequential) [17] weight reductions of only 1.5 and 1.3 kg, respectively, relative to the control intervention consisting of generic, brief, structured dietary modification advice [33]. In aggregate, these results are similar to our findings and suggest that the effectiveness of more intensive self-management interventions, whether delivered in a web-based or in-person format, is likely to be limited relative to providing simple and standardized printed educational materials to patients. One important difference between this study and prior trials is that we conducted a pragmatic evaluation of interventions that have been implemented and are currently in use in clinical practice; thus, our results depict a more realistic assessment of what can be expected outside of a highly selected clinical trial sample.

Strengths of this study include its pragmatic comparative effectiveness approach, its randomized design and its assessment of a broad range of endpoints important to patients, providers and decision makers. There are also several limitations. First, nearly 30% of individuals withdrew early, which is a common finding in trials enrolling subjects with severe obesity and thus not unexpected [34, 35]. Accordingly, we employed a conservative baseline-observation-carried forward approach, chosen a priori, for the primary analysis. In addition, for weight-related outcomes, we reported a less conservative on-treatment analysis, and these results were very similar to those of the primary analysis, demonstrating that our findings were robust relative to the type of analytic strategy used. Second, we examined a population with severe obesity being considered for bariatric surgery, and our results may have been different if we had examined a primary care population with lesser degrees of obesity. Third, patients assigned to web-based modules may have logged in but not reviewed the content, limiting effectiveness. Fourth, there may be concerns related to external validity, with our results most specific to the Northern and Central Alberta multidisciplinary bariatric specialty care programs. That said, the populations studied and the types of programs evaluated should be generalizable to many other Canadian bariatric programs, particularly because many of these programs were based on the Edmonton Weight Wise model [17]. By the same token, further generalization to similar publicly funded programs in other countries may be reasonable but should be made with caution. In fact, our findings suggest it is important to undertake rigorous evaluation before widespread adoption, as our experience (and that of others) [36] suggests that ’de-implementation’ is extremely difficult.

## Conclusion

In conclusion, the EVOLUTION trial demonstrated that more intensive and costly in-person or web-based self-management interventions were no more effective than the provision of printed educational materials for patients with severe obesity. Given these findings and the perpetual scarcity of health care funds, we believe the resources currently used to deliver in-person and web-based programs should be redeployed towards developing (and testing) more effective interventions rather than maintaining the status quo.

## Additional file

Additional file 1: Table S1. Weight Wise Community Module: in-person program. **Table S2.** Weight Wise Community Module: web-based program. **Table S3.** Health care provider visit frequency by study arm. (DOCX 25 kb)
