# Supplementary material for: The evaluating self-management and educational support in severely obese patients awaiting multidisciplinary bariatric care (EVOLUTION) trial: principal results
Source: BMC Med. 2017 Mar 2;15:46. doi: 10.1186/s12916-017-0808-6 (PMC5333420; doi:10.1186/s12916-017-0808-6)
Supplement: Additional file 1: Table S1. — Weight Wise Community Module: in-person program. Table S2. Weight Wise Community Module: web-based program. Table S3. Health care provider visit frequency by study arm. (DOCX 25 kb) [file 12916_2017_808_MOESM1_ESM.docx]

**Additional file 1**

**Table S1. Weight Wise Community Module*: In-person Program**

| **Topic** | **Content** |
| --- | --- |
| **Module One - Getting Started: Planning for Success** | Learn about obesity – definition, causes and treatment options including bariatric surgery.  Learn about how obesity impacts your life and health.  Learn ways to record what you eat, how active you are and how you are feeling emotionally.  . |
| **Module Two – Lifestyle Change: A Toolkit for Success** | Learn how to make and keep healthy lifestyle changes.  Learn strategies such as prioritizing, journaling, problem solving, thinking skills and goal setting. |
| **Module Three - Finding Balance: The Role of Calories in Weight Management** | Learn about the top 5 ways to lower calories and practice strategies in class. |
| **Module Four - Managing Hunger and Appetite** | Explore the difference between hunger and appetite.  Practice techniques to manage appetite triggers and social pressures. |
| **Module Five - Moving Matters – Including Physical Activity in Your Day** | Explore the benefits and barriers to being more active.  Receive tips on how to get more physically active.  Set your own personal activity goals. |
| **Module Six - Nutrition: The Truth About What Works in Weight Management** | Evaluate what you are eating and learn strategies that can help lower your calories.  Explore how meal patterns, food choices and portion size affect calorie intake.  Learn about which foods can help you manage your weight. |
| **Module Seven - Nutrition: I Know I Should Eat Healthy, But How?** | Leave with tips on how to put your nutrition knowledge into practice.  Explore the 4 P’s: **P**lan, **P**urchase, **P**repare and **P**ack. |
| **Module Eight- Nutrition: Eating Away From Home and During Special Occasions** | Learn how buffets, parties, vacation and holiday eating can affect calorie intake.  Leave with strategies to minimize extra calories when eating away from home and during special occasions. |
| **Module Nine - Minding Stress: Effectively Reduce and Manage the Stress in Your Life (two consecutive sessions)** | Discover the hidden costs of stress; explore what causes you stress and what you can do about it.  Opportunity to learn about and experience effective stress-management techniques  Uncover the world of Mindful Eating |
| **Module Ten - Managing Emotional Eating (3 Session Series)**  **(Edmonton only)** | Learn about what influences your eating behaviors  Build skills and learn tools to help you manage your emotional eating |

**Table S2. Weight Wise Community Module: Web-based Program**

| Getting Started with Weight Management |
| --- |
| Weight Management Benefits and Challenges |
| Skills for Weight Management Success |
| Calories and Diets- What Works, What Doesn’t |
| Top 5 Calorie Culprits |
| Transform Your Eating for Weight Management |
| Meal Planning Tips and Label Reading |
| Eating Out and Managing Weight |
| Managing Your Hunger |
| How to Control Your Appetite |
| How Does Moving Matter? |
| I’m Ready to Get Moving! Help Me! |
| A Good Night’s Sleep |

**Table S3.** Health Care Provider Visit Frequency by Study Arm

| **Health Care Provider** | **In-person**  **Mean no. of visits±SD** | **Web-based**  **Mean no. of visits±SD** | **Controls**  **Mean no. of visits±SD** | **p-value*** |
| --- | --- | --- | --- | --- |
|  | **n=182** | **n=200** | **n=196** |  |
| Dietician | 2.4±1.5 | 2.4±1.4 | 2.3±1.6 | 0.85 |
| Physician | 1.4±1.2 | 1.3±0.9 | 1.4±1.3 | 0.61 |
| Sleep Specialist | 0.6±1.0 | 0.5±0.9 | 0.5±0.9 | 0.35 |
| Psychologist | 2.2±2.5 | 2.1±2.4 | 1.9±2.3 | 0.51 |
| Psychiatrist | 0.4±0.8 | 0.4±0.7 | 0.4±0.8 | 0.66 |
| Occupational therapist | 0.03±0.21 | 0.05±0.24 | 0.05±0.23 | 0.75 |
| Physiotherapist | 0.04±0.29 | 0.01±0.14 | 0.04±0.31 | 0.47 |
| Social Worker | 0.02±0.15 | 0.03±0.19 | 0.03±0.19 | 0.97 |

Nurses act as case-managers to coordinate the patient visits. Their visit frequency was not tracked.

*using one-way analysis-of-variance; SD=standard deviation
